# The Application of Metabolomics for the Study of Cereal Corn (*Zea mays L.*)

**DOI:** 10.3390/metabo10080300

**Published:** 2020-07-23

**Authors:** Lena Gálvez Ranilla

**Affiliations:** Laboratory of Research in Food Science, Universidad Catolica de Santa Maria, Urb. San Jose s/n, 04013 Arequipa, Peru; lgalvez@ucsm.edu.pe; Tel.: +51-54-382038

**Keywords:** *Zea mays* L., metabolomics, genetic diversity, secondary metabolites, crop improvement

## Abstract

Corn (*Zea mays* L.) is an important cereal crop indigenous to the Americas, where its genetic biodiversity is still preserved, especially among native populations from Mesoamerica and South America. The use of metabolomics in corn has mainly focused on understanding the potential differences of corn metabolomes under different biotic and abiotic stresses or to evaluate the influence of genetic and environmental factors. The increase of diet-linked non-communicable diseases has increased the interest to optimize the content of bioactive secondary metabolites in current corn breeding programs to produce novel functional foods. This review provides perspectives on the role of metabolomics in the characterization of health-relevant metabolites in corn biodiversity and emphasizes the integration of metabolomics in breeding strategies targeting the enrichment of phenolic bioactive metabolites such as anthocyanins in corn kernels.

## 1. Introduction

Corn or maize (*Zea may* L. ssp. *mays*) is indigenous to the Americas and is one of the most important cereal crops worldwide [1]. Corn is a staple food in many countries from Mesoamerica, South America, and Africa and also has a deep ethnic and cultural relevance among indigenous populations in these regions. In addition, this cereal is also used to produce feed, fuel, and as a raw material for diverse products at industrial level. Global corn production was about 1147 million tons in 2018, and the major producing countries were the United States, China, and Brazil [2].

Corn was domesticated from the wild annual teosinte (*Zea mays* spp. *parviglumis*) around 9000 years ago in Mexico in a single event [3]. The domestication process involved the adaptation of corn from its tropical origin to temperate and other diverse environmental conditions, undergoing a significant morphological transformation in the plant and the inflorescence architecture [4]. The primitive and early improved corn was spread across North and South America and adapted to new environments and uses, leading to the origin of “landraces or races” [5]. Landraces are highly heterozygous and heterogeneous open-pollinated populations [5]. Anderson and Cutler [6] first proposed the definition of “race” as “a group of related individuals with enough characteristics in common to permit their recognition as a group.” Salazar et al. [7] defined landraces or local races as the germplasm that has been cultivated for hundreds of years under traditional agriculture systems and which represent a high genetic diversity. This material has been generally selected by native farmers for better adaptation to specific environments, yield, nutritive value, cultural use, and resistance to biotic and abiotic stresses [8]. Landraces are dynamic populations that lack formal crop improvement and are genetically diverse [9]. With the advent of Europeans to America, corn was introduced to different continents including Europe, Africa, and Asia, following variable patterns of adaptation and breeding [10,11]. The advance of more modern breeding techniques has allowed the production of high-yielding inbred corn lines (made by self-pollination) from landraces, and then hybrids or commercial varieties have been obtained based on crossed inbred lines. The current corn germplasm is often referred to in terms of teosinte, landraces, and inbred lines and are important genetically diverse resources for crop improvement and food security [5].

The current challenge for feeding a growing population that would almost reach 10 billion over next 30 years is creating the need to improve existing crops and develop new crops with even higher yielding, more nutritious, and with climate change resilient characteristics [12]. On the other hand, the rise of diet-linked chronic diseases worldwide, such as type 2 diabetes, and other derived complications including cancer and certain degenerative diseases has increased the interest in plant-derived foods from global biodiversity [13,14,15]. Fruits and whole grains have been found to be sources of bioactive compounds with potential to decrease the risk of chronic diseases when consumed on a regular basis. The intake of whole grains rich in different health-relevant functional metabolites has been related to the reduction of risks factors of major chronic diseases according to several epidemiological and intervention studies [16,17,18]. The research and sustainable use of corn genetic diversity may help to counter such challenges.

Metabolomics has emerged as a valuable technology for the comprehensive profiling and comparison of metabolites in biological systems, and a diversity of applications in plant sciences have been reported [19,20]. Depending on the purpose of the study and the type of scientific information required, metabolomics may be focused into different approaches such as targeted analysis, metabolomic profiling and metabolomic fingerprinting [21,22]. In the case of corn, several metabolomic studies have focused on understanding the complex biochemical mechanisms involved in corn plant response to environmental directed biotic [23,24,25,26,27] and abiotic stress factors [28,29,30,31,32]. Other metabolomic studies have been aimed at comparing genetically modified and non-genetically modified corn lines [33] or at evaluating the influence of genetic factors and the growing location on corn kernel and plant function [34,35,36,37].

The current review summarizes the application of metabolomics for the characterization of health promoting metabolites in corn kernels and emphasizes the importance of corn genetic diversity, especially native landraces, as potential sources of bioactive metabolites. The role of metabolomics integrated to novel phenotype-genotype studies targeting the improvement of health-relevant metabolites in corn with a focus on anthocyanins is also presented. Future perspectives on the application of metabolomics for the valorization and understanding of corn genetic diversity and its impact on food security and health are also highlighted.

## 2. The Diversity of Corn Germplasm and the Importance of Native Landraces

The preservation of genetic resources including crop landraces and wild relatives is fundamental and crucial for future crop improvement [38]. Landraces have been found to possess important allelic diversity contributing, for example, with useful genes for a more efficient plant nutrient uptake and utilization, resistance or tolerance to biotic and abiotic stresses, and superior nutritional and health-linked bioactive compounds [39].

**Figure 1:**
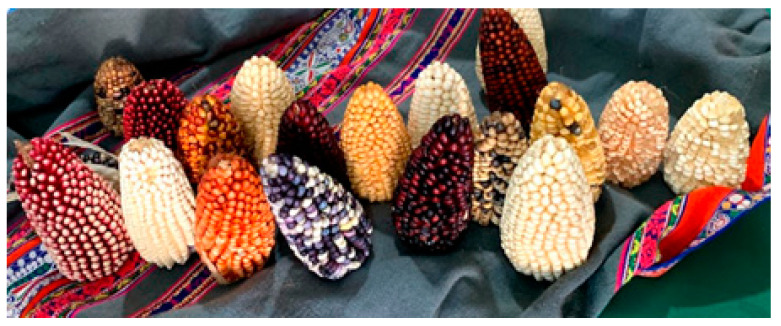
Phenotypic variability of some corn landraces from Southern Andean region of Peru (from author’s personal file).

Ex situ and in situ strategies have been applied to conserve corn genetic diversity worldwide. The first international initiative for the ex situ conservation of corn germplasm was directed by the Latin American Maize Program (LAMP) (1987–1996). A group of 11 countries from Latin America and the United States evaluated the status of local corn germplasm, compiling information about this germplasm, and promoted the access to this information for research purposes and for the creation of superior varieties and hybrids by breeders [40]. Table 1 shows the genetic diversity of corn germplasm in terms of the number of native landraces reported by country in the Americas and in some countries from different continents. Corn genetic diversity is very high in Latin America, and there are around 300 landraces that comprise 90 percent of world’s corn diversity [40,41]. Mexico and Peru have around 30 percent of Latin American corn landrace diversity. The phenotypic and molecular variability of corn landraces from Mexico, the center of corn origin, has been reported by Prasana [8] and Prasana [42]. The Andean region has wide ecological diversity and corn landraces that are currently grown from areas at sea level to different highland regions. These adaptations have been developed by native farmers since ancient times. This may explain the fact that the Andean region is possibly the geographical area with the highest corn phenotypic diversity in the world (Figure 1) [43]. However, Andean corn landraces have been poorly investigated at phenotypic and molecular levels compared to the research focused on Mexican landraces and teosinte varieties.

In several places in Mesoamerica and Latin America, local farmers maintain the cultivation of corn landraces for subsistence under traditional agriculture practices (in situ conservation). However, the increasing introduction of modern corn hybrids is leading to a steady genetic erosion of native germplasm. This situation will compromise food security, potentially affecting indigenous food systems in the medium term and potentially long term.

Germplasm banks from the International Maize and Wheat Improvement Center (CIMMYT—Mexico City, Mexico) and the United States Department of Agriculture-National Plant Germplasm System (USDA-NPGS—Washington, DC, United States) currently preserves the world’s major corn collections [61,62]. However, modern breeding programs are mainly based on corn germplasm with narrow genetic base as was reported by Prasanna [8]. Considering the current demand of healthier plant-based foods due to the increase of metabolic chronic diseases worldwide, the integrated research of native corn landraces may increase the genetic base for crop improvement and targeting functional bioactive metabolites beyond yield and common agronomic traits.

## 3. The Application of Metabolomics for the Characterization of Health-Relevant Metabolites in Corn Genetic Diversity

Plant secondary metabolites are small molecules that are considered non-essential for the general survival of the organism but are key components for overall plant adaptation and protection to biotic and abiotic stress conditions [63]. Several epidemiological and intervention studies have shown an inverse correlation in the prevalence of major chronic diseases including cardiovascular disease, type 2 diabetes, and cancer and the consumption of whole grains [16,17,64]. This is due to the presence of a wide range of phytochemical compounds, mainly secondary metabolites, that also play a significant role for human health [64].

The composition of corn in terms of primary metabolites with major nutritional importance (proteins, carbohydrates, fat) along with the mineral composition has been shown to be comparable or even better than common cereal crops such as rice and wheat [65]. Some comprehensive reviews have also shown that the corn kernel is a good source of important health-relevant phytochemicals such as phenolic compounds, carotenoids, tocopherols, and phytosterols [65,66]. However, research on the phenolic and carotenoid composition in corn kernel has received more attention due to the wide chemical diversity and remarkable health-promoting properties associated to both metabolite groups [67,68].

### 3.1. Metabolomic Analysis of Phenolic Compounds in Corn Genetic Diversity

The characterization of phenolic bioactives in corn kernel diversity was first evaluated by applying spectrophotometric methods on Mexican landraces of variable kernel pigmentation [69,70,71,72]. This has allowed the rapid selection of promising corn accessions and genotypes for further breeding strategies. The specific chemical structure of phenolic compounds has been associated with different functional properties based on their antioxidant capacity [73]. In addition, phenolic compounds in cereals such as corn can be classified into free, esterified (covalently bound to other molecules), and insoluble-bound forms. Insoluble-bound phenolics form covalent bonds with cell wall macromolecules including pectin, cellulose, arabinoxylan, and structural proteins and generally represent the major phenolic fraction in cereals [74]. Bound phenolics are not significantly affected by the gastrointestinal digestion but are released through colonic fermentation exerting a myriad of positive biological effects at colon level [75].

The study of phenolic profiles (free and bound phenolic fractions) in corn (kernel) germplasm from different origins and types (landraces, inbred lines, hybrids, teosinte varieties) has been done using mainly targeted metabolomic platforms (Table 2 and Table 3). Corn landraces and hybrids with different phenotypes (kernel color) from American germplasm banks (Mexico and the United States) have been extensively screened as important sources of phenolic compounds through the use of a liquid chromatography diode array detector/ultraviolet-visible detector (LC-DAD/UV-Vis) and liquid chromatography coupled to mass spectrometry (LC-MS). The interest in the characterization of high-anthocyanin corn landraces and hybrids is increasing due to the growing demand for alternative sources of natural pigments for nutraceutical and food industry applications. Hong et al. [76] and Hong et al. [77] have recently combined the use of different mass separation techniques such as quadrupole-Orbitrap and triple quadrupole for the detection of at least 18–20 different types of anthocyanins in purple corn germplasm from Australia. These authors optimized the method for the extraction and detection of anthocyanins, revealing that some previously identified anthocyanins in purple corn such as succinyl and ethyl-malonyl derivatives are likely not to be endogenous but are esterification products formed during the extraction process [76,77].

The most comprehensive survey for the identification of high-anthocyanin corn germplasm was done by Paulsmeyer et al. [78]. Around 398 genetically diverse pigmented corn accessions from different origins were analyzed, and 167 accessions were selected due to their capability to produce anthocyanins. Selected germplasm was classified as blue aleurone, pink aleurone, purple pericarp with condensed anthocyanin forms, purple pericarp without condensed forms, and reduced acylation accessions. Accessions with purple pigmented pericarp with the presence of condensed forms of anthocyanins showed the highest anthocyanin contents (a Peruvian Andean purple corn had the highest levels). The same study also revealed that the anthocyanin biosynthesis has a broad potential of heritability, and this trait can be optimized through breeding strategies [78].

Peniche-Pavia [79] recently showed the potential of direct injection electrospray ionization mass spectrometry (DIESI-MSQD) as a cost-effective, more economical, and statistically robust method for high-throughput phenotypic characterization and targeting of high anthocyanin corn germplasm. Furthermore, Fourier-transformed near-infrared reflectance spectroscopy (FT-NIRS) has been used for the quantitative measure of anthocyanin contents in a wide array of corn germplasm [80]. Vibrational spectroscopy (infrared and NIR) includes non-destructive methods that can provide high-throughput analysis of a large number of samples with minimum sample preparation. However, major drawbacks are their poor resolution and limited information about the chemical structure of targeted metabolites [81].

### 3.2. Metabolomic Analysis of Carotenoid Compounds in Corn Genetic Diversity

Carotenoids are natural pigments found in most fruits, vegetables, and grains. These metabolites have shown a range of beneficial functional properties for human health. Besides their antioxidant effects, some individual carotenoids such as β-carotene, β-cryptoxanthin, and other derivatives have a pro-vitamin-A function [68]. Lutein and zeaxanthin are essential for macular pigments in the eye and have been linked to the reduction of the macular eye disease reducing the risk of cataracts [97]. LC coupled to detectors such as ultraviolet-visible (UV-Vis) and diode array detectors (DADs) have been widely used for the identification of pro and non-provitamin A carotenoids in different corn germplasm (Table 4). Liquid chromatography tandem mass spectrometry (LC-MS/MS) has allowed the generation of more information about the variability of carotenoid isomers present in corn kernels [98].

In contrast to the observations on corn phenolic compounds, several investigations from different continents have focused their attention on the characterization of carotenoids in worldwide corn biodiversity. Corn has been targeted as one of the major crops for provitamin A enrichment, and important global initiatives such as the HarvestPlus and CYMMIT programs have focused on obtaining high-provitamin-A corn cultivars through conventional and molecular breeding strategies [99,100]. The main objective of the HarvestPlus program (2003–2016) was to develop high-yielding provitamin-A-enriched corn cultivars with proven efficiency in reducing vitamin A deficiency that is profitable to farmers and acceptable to the consumers [100,101]. Targeted metabolomics have revealed that carotenoids are mostly concentrated in the endosperm and that overall orange-pigmented varieties show the highest carotenoids levels (Table 4). This germplasm has potential for applications in biofortification-linked breeding programs.

### 3.3. Use of Non-Targeted Metabolomic Platforms for the Research of Corn Kernel Metabolome

Untargeted metabolomics alone or integrated to other high-throughput platforms such as transcriptomics and proteomics have been recently used to characterize the composition of corn kernels and to understand the molecular regulation of metabolic pathways underlying the biosynthesis of phenolic antioxidants. Rao et al. [112] identified 210 metabolites (199 primary metabolites, nine secondary metabolites, and two phytohormones) in mature kernels of 14 corn lines from China using LC-MS/MS and gas chromatography mass spectrometry (GC-MS). A total of 32 metabolites were identified corresponding to basic and essential macronutrients (17 amino acids, five carbohydrates, seven lipids, and three cofactors, prosthetic groups, electro carriers). Other molecules such as vitamin E, stigmasterol, campesterol, β-sitosterol, phospholipids, antioxidants (dihydro-kaempferol and the lactone costunolide), and the antinutrient inositol hexaphosphate were also detected. An integrated metabolic map based on transcriptomic, proteomic and metabolomic data was built and included seven important pathways and 23 sub-pathways that represented the regulatory mechanisms of corn kernel metabolism [112].

Hu et al. [113] studied the metabolome of purple pigmented and non-pigmented kernels from China at different maturity stages (11, 16, 21 days after pollination (DAP)) using a combination of LC-MS/MS and GC-MS. A total of 247 metabolites were identified and metabolomes differed according to the maturity stage. The integration of transcriptomic data revealed that kinetic trends in the transcriptome and metabolome were similar across kernel development. An important metabolic shift from the primary to the secondary metabolism occurred at 16 DAP only in the purple pigmented kernel targeting the biosynthesis of anthocyanins and precursors of phlobaphenes [113]. The biochemical information generated in above studies would be valuable in molecular breeding strategies aimed at increasing health promoting metabolites such as anthocyanins in corn kernel.

Unlike the high-throughput analytical platforms such as LC/GC-MS for metabolomic studies, mass spectrometry imaging (MSI) is a relatively recent non-targeted method used for the simultaneous analysis of both the composition and spatial distribution of many compounds [114]. MSI is a two-dimensional analysis method that can detect intact molecules within tissue sections without requiring extraction, purification, separation, or labeling while allowing the detection of a wide range of molecules [114,115]. MSI has the capability to image thousands of metabolites such as lipids, peptides, proteins, and secondary metabolites (including phenolic compounds) in a single experiment [116,117,118]. Most common MSI configurations are matrix-assisted laser desorption ionization mass spectrometry (MALDI-MSI), secondary ion mass spectrometry (SIMS), and desorption electrospray ionization mass spectrometry (DESI), depending on the type of ionization [114]. In case of corn, MSI has been applied for the study of kernel tissue sections, the distribution of lysophospholipids within starch granule in corn endosperm, and the distribution of cellulose and hemicellulose structures in corn stems [119,120,121]. MSI can provide valuable information about the localization of important bioactive compounds such as anthocyanins for breeding purposes. The integration of these untargeted platforms with targeted methods might help to increase the analytical power for the comprehensive characterization of corn kernel diversity.

## 4. Role of Metabolomics in Corn Molecular Breeding Strategies Targeting Health-Relevant Phenolic Metabolites

### 4.1. Common Methods Used for the Study of Corn Genetic Diversity

Genetic diversity has been broadly defined as any variation in the nucleotides, genes, chromosomes, or genomes of a species at a level of the individual, population, species, or region for a given time [122]. This diversity is highly desirable in crop breeding programs since it offers a wide array of critical gene pools for further development of improved corn seeds relevant for nutrition and health. As mentioned above, corn genetic diversity (native landraces) may represent an important source of candidate genes for breeding applications.

The study of genetic diversity in corn germplasm has been assessed using different types of markers (Table 5). Morphological markers such as those related with plant traits (genetically determined characteristics) [5] with agronomic interest have been traditionally used in several studies for the elucidation of potential clustering patterns among different corn landraces. Ortiz et al. [123] and Ortiz et al. [124], using plant and internal ear traits, respectively, determined that several Andean corn accessions can be classified in different groups corresponding mainly to the original corn race classification. Likewise, Salazar et al. [125] highlighted the wide range of phenotypic variability based on different plant qualitative and quantitative traits at an intra-racial level in the Chilean Choclero landrace. The genetic diversity of Turkish corn genotypes can be classified into flint, pop, and dent races using ear and tassel traits [126].

Other authors have used molecular markers such as microsatellites (SSR) to assess the genetic diversity in germplasm from Brazil and Argentina [127,128]. Porta et al. [129] confirmed the genetic diversity and representativeness of the core collection (90 accessions) with respect to the whole Uruguayan corn landrace collection applying SSR markers in combination with morphological characteristics. The advance in the development of high-throughput sequencing techniques have allowed the genotyping-by-sequencing (GBS) of 349 commercial corn inbred lines based on single-nucleotide polymorphism (SNP) markers [130]. Same study revealed the presence of three different heterotic groups corresponding to the Stiff Stalk, Non-Stiff Stalk, and Iodent corn types among North American corn germplasm [130].

### 4.2. Metabolomic-Assisted Molecular Breeding Strategies for the Increase of Phenolic Antioxidants in Corn Kernels

Metabolomics enables the comprehensive high-throughput quantification of a wide array of metabolites and is becoming an important tool for corn phenotyping. Since the metabolic phenotype provides a link between gene sequence and visible phenotypes, metabolites can be used as markers for trait prediction relevant for crop genetic improvement [132,133]. Strategies known as association mapping studies including genome-wide association (GWAS) and linking studies have been developed for the identification of genes underlying quantitative trait loci (QTL) (a region of the genome associated with the control of a quantitative trait) [5,134,135]. GBS profiling combined with different omics technologies are currently applied to understand the genetic and biochemical regulation of metabolism linked to relevant agronomic and nutritional traits in corn.

Secondary metabolites such as anthocyanins are receiving more attention for breeding purposes due to their well-known health-relevant bioactivity and potential for use as natural pigments at the industrial level. Metabolite-based genome-wide association studies (mGWAS) are used to decipher the genetic basis of plant metabolite biosynthesis and regulation. This strategy is allowing the development of metabolic markers and genetic loci for metabolome-assisted biofortification [136]. The anthocyanin biofortification through mGWAS techniques has already been obtained in tomato and rice [137,138]. Rhodes et al. [139] evaluated a wide sorghum diversity panel (381) using NIRS and identified novel QTL for sorghum polyphenols through a GWAS with 404,628 SNP markers.

Conventional marker-assisted breeding has been used for the production of a colored polenta corn kernel and a purple popcorn rich in anthocyanins in Italy [140,141]. GWAS strategies have been more extensively used for the study of complex traits, such as yield and stress resistance in corn plants [142]. Novel metabolites and genes involved in the biosynthesis of flavonoids have been identified in mature kernels from a diverse corn inbred panel (368) through mGWAS using different LC-MS/MS platforms (electro spray ionization-triple quadrupole-linear ion trap and electro spray ionization-high-resolution quadrupole time-of-flight mass spectrometry, respectively) [133]. However, the application of mGWAS targeting the biofortification of corn kernel in relation to anthocyanin metabolites have not been well studied. This technique along with other new GWAS methods and new population designs would accelerate the discovery and use of allelic diversity in corn landraces to produce novel corn-based functional foods [12,142].

## 5. Future Perspectives

Metabolomics is an important technique for the characterization of relevant bioactive phytochemicals such as phenolic and carotenoids compounds in corn biodiversity. The integration of metabolomic information with data from other high-throughput omics technologies is critical for better understanding the underlying molecular mechanisms involved in the biosynthesis of functional metabolites. The application of novel phenotype-genotype molecular strategies for corn breeding such as mGWAS would speed up the production of innovative functional corn-derived foods with improved health-relevant compounds such as anthocyanins and carotenoids.

Corn landrace genetic diversity is a key source of candidate genes with potential applications in modern breeding programs aimed at producing healthier foods in resilient and sustainable ecosystems. A primary major challenge is the comprehensive molecular characterization of worldwide corn landrace diversity using an integrated multi-omics approach including metabolomics. A high-quality phenotypic data is essential to identify and use relevant corn germplasm for different breeding applications.

Extensive research has focused on corn diversity from North and Mesoamerica as highlighted in this review. The Andean region also represents an important hotspot of corn diversity, but the scientific information about germplasm from this area is limited. In fact, some breeding initiatives based on Peruvian corn with high anthocyanin contents have been carried out in the United States and Australia [77,78]. The effective in situ and ex situ preservation of corn diversity at local and international levels is critical to ensure adequate research on corn germplasm, especially in areas with high corn genetic diversity. This requires clear rules and policies for its accession and exchange involving not only researchers but also critical stakeholders such as farmers, government, and industry.

The next challenge that requires a multidisciplinary and intersectoral approach is the transition from the laboratory or greenhouse to the field. All strategies should consider the specific environmental conditions of the target region for corn cultivation. Applications in geographical areas where corn diversity is high may be strategic for improving corn in situ conservation while allowing the development of local agriculture within indigenous food systems.

The holistic integration of metabolomics with complementary omic-technologies may open the opportunity for the application of omic-wide association studies targeting the valorization, understanding, and sustainable use of worldwide corn genetic diversity.

## Figures and Tables

**Table 1:** Number of native corn landraces per country (ex situ conservation).

Geographical Area	Country	Nº Landraces	Nº Accessions in Local Germplasm Bank
America	Argentina	43 [44]/51 [45]	1927 [44]/2035 [46]
	Bolivia	45 [47]	>1000 [47]/1080 [46]
	Brazil	27 [48]	1743 [46]
	Chile	23 [49]	929/945 [46]
	Central America	20 [50]	1231 [50]
	Colombia	23 [46]	2050 [46]
	Ecuador	29 [51]	532 [46]
	Mexico	59 [46]	6738 [46]
	Peru	49 [52]/52 [53]	3023 [46]
	Paraguay	10 [54]	478 [46]
	United States	9 [55]	900 [46]
	Venezuela	19 [56]	724 [46]
Europe	Italy	34 [57]	562 [57]
	Portugal	10 [58]	
	Yugoslavia	18 [59]	˜2000 [59]
Asia	India	15 [60]	1300 [60]

**Table 2:** Targeted metabolomics using liquid chromatography mass spectrometry (LC-MS) platforms for the characterization of phenolic bioactive compounds in worldwide corn biodiversity.

Geographical Area	Type of Sample	Germplasm Origin (Place/Bank)	Analytical Configuration	Phenolic Metabolite in Mature Corn Kernels		Relevant Germplasm	Reference
				Free Fraction	Bound Fraction		
America	Ten landraces: (purple) Kulli, Ayzumo, Paru, Tuimuru, Oke, Huaca, Songo, Colorado, Huillcaparu, Checchi	Bank: Centro de Investigaciones Fitoecogenéticas de Pairumani (Cochabamba, Bolivia)	HPLC-ESI- MS/MS (IT) ^1^	Phenolic acids: *p*-coumaric acid, ferulic acid Anthocyanins (10): Cy3G ^12^, Cy3MG ^13^, Cy3DMG ^14^ Pg3G ^15^, Pg3MG ^16^, Pg3DMG ^17^ Pn3G ^18^, Pn3MG ^19^, Pn3DMG ^20^ Epicatechin-Cy3,5DG ^21^	Phenolic acids: *p*-coumaric acid, ferulic acid, ferulic acid dehydrodimers (8-5’diferulic acid), ferulic acid dehydrotrimers.	Kulli (high anthocyanin contents)	[82]
	Twenty-three landraces: (colored and uncolored) with a Peruvian purple corn as a control	Departments: Santander (4), Cundinamarca (7), Boyacá (12) (Colombia)	HPLC-UV-DAD-ESI-MS ^2^	Phenolic acids: caffeic acid, chlorogenic acid, caffeic acid derivative, ferulic acid derivative, sinapic acid derivative. Flavonols: isoquercetin, astragalin, quercetin-rutinoside, isorhamnetin-glucoside, quercetin-diglucoside. Anthocyanins (9): Cy3G ^12^, Cy3MG ^13^, Cy3SG ^22^, Cy3EMG ^23^ Pg3G ^15^, Pg3MG ^16^, Pg3DG ^24^ Pn3G ^18^, Pn3MG ^19^.		Purple colored samples (high phenolic contents)	[83]
	Eight accessions (landraces and open pollinated varieties): Navajo Blue, Santa Clara Blue, Flor del Rio, Yoeme Blue, Hopi Blue, Taos Blue, Ohio Blue (Corn Belt dent), Los Lunas High	Bank: Native Seeds/SEARCH (AZ, United States) Bank: Plants of the Southwest (NM, United States)	HPLC-DAD-MS ^3^ HPLC-MS (FTICR) ^4^	Anthocyanins (5): Cy3G ^12^, Cy3SG ^22^, Cy3DSG ^25^ Pg3G ^15^ Pn3G ^18^		Navajo Blue and Ohio Blue (high anthocyanin contents)	[84,85]
Oceania	Six varieties: purple-pericarp sweet corn, reddish-purple-pericarp sweet corn, purple-pericarp, corn, purple-pericarp-blue-aleurone corn, blue aleurone, cherry-aleurone corn	Gatton Research Facility (QLD, Australia)	UHPLC-DAD ^5^ UHPLC-MS/MS (Q-Orbitrap) ^6^ UHPLC-ESI-MS/MS (QQQ) ^7^	Anthocyanins (18): Cy3G ^12^, Cy3MG ^13^ (4 isomers), Cy3DMG ^14^ (3 isomers). Pg3G ^15^, Pg3MG ^16^ (2 isomers), Pg3DMG ^17^ (2 isomers). Pn3G ^18^, Pn3MG ^19^ (3 isomers), Pn3DMG ^20^		Purple pericarp-corn (high anthocyanin contents)	[76]
	Two varieties (derived from PB12.5-2-1 line and a purple Peruvian corn 9PW1): Purple-pericarp and reddish-purple pericarp “super sweet”			Anthocyanins (20): Cy3G ^12^, Cy3MG ^13^ (4 isomers), Cy3DMG ^14^ (3 isomers). Pg3G ^15^, Pg3MG ^16^ (4 isomers), Pg3DMG ^17^ (3 isomers). Pn3G ^18^, Pn3MG ^19^ (2 isomers), Pn3DMG ^20^		Purple-pericarp corn	[77]
Asia	Twelve waxy colored corn genotypes (commercial and landraces)	Bank: Plant Breeding Center for Sustainable Agriculture (Khon University, Thailand). Genotypes from various countries of Asia: Thailand, Korea, China, Laos, Taiwan.	HPLC-DAD-MS ^3^	Anthocyanins (10): Cy3G ^12^, Cy3MG ^13^, Cy3DMG ^14^, Cy3SG ^22^, Cy3MSG ^26^ Pg3G ^15^, Pg3MG ^16^. Pn3G ^18^, Pn3MG ^19^, Pn3SG ^27^		Purplish black genotype KKU-WX111031 (high anthocyanin contents)	[86]
	Five corn hybrids: Jingke 968, Zhengdan 958, Xianyu 335, Jingkenuo 2000, Jingketion 183	Bank: Beijing Academy of Agriculture and Forestry Sciences (Beijing, China)	UPLC-MS/MS (QTOF) ^8^	Flavanones: eriodyctiol, luteolin		Jingke 968 hybrid (high phenolic contents)	[87]
	Four corn hybrids: HQPM-7 (quality protein maize), HM-4 (baby corn), VL Amber (popcorn), Madhuri (sweet corn)	Bank: Directorate of Maize Research (New Delhi, India)	HPLC-DAD ^9^ LC-ESI-MS/MS (QTOF) ^10^	Phenolic acids: *p*-hydroxybenzoic acid, vanillic acid, syringic acid, caffeic acid, *p*-coumaric acid, ferulic acid, iso-ferulic acid. Anthocyanins: Cy3G ^12^ Flavonols: kaempferol, quercetin.	Phenolic acids: *p*-hydroxybenzoic acid, vanillic acid, syringic acid, caffeic acid, *p*-coumaric acid, ferulic acid, iso-ferulic acid. Anthocyanins: Cy3G ^12^ Flavonols: kaempferol, quercetin.	Madhuri (sweet corn) (high phenolic contents)	[88]
Mixed	398 genetically diverse pigmented corn accessions from different origins	Banks: NCRPIS (North Central Regional Plant Introduction Station, Ames, United States). CIMMYT (International Maize and Wheat Improvement Center, Mexico). Native Seeds/SEARCH (AZ, United States). MGCSC (Maize Genetics Cooperation Stock Center, IL, United States). Commercial sources.	HPLC-UV-VIS ^11^ LC-MS	Anthocyanins (9-10): Cy3G ^12^, Cy3MG ^13^ Pg3G ^15^, Pg3MG ^16^, Pg3DMG ^17^ Pn3G ^18^, Pn3DMG ^20^ Cy3DMG ^14^/Pn3MG ^19^ (coeluted) Flavanol-anthocyanin dimers (condensed anthocyanin forms)		167 accessions (with anthocyanin production) Peruvian landrace: Arequipa 204 (PI571427) (high anthocyanin contents)	[78]

^1^ High-performance liquid chromatography electro spray ionization ion trap tandem mass spectrometry. ^2^ High-performance liquid chromatography ultraviolet-diode array detector electro spray ionization-mass spectrometry. ^3^ High-performance liquid chromatography diode array detector mass spectrometry. ^4^ High performance liquid chromatography Fourier-transformed ion cyclotron mass spectrometry. ^5^ Ultra-high-performance liquid chromatography diode array detector. ^6^ Ultra-high-performance liquid chromatography quadrupole orbitrap tandem mass spectrometry. ^7^ Ultra-high-performance liquid chromatography electro spray ionization triple quadrupole tandem mass spectrometry. ^8^ Ultra-performance liquid chromatography quadrupole time of flight tandem mass spectrometry. ^9^ High-performance liquid chromatography diode array detector. ^10^ Liquid chromatography electro spray ionization-quadrupole time of flight tandem mass spectrometry. ^11^ High-performance liquid chromatography ultraviolet-visible detector. ^12^ Cyanidin-3-glucoside. ^13^ Cyanidin-3-malonylglucoside. ^14^ Cyanidin-3-dimalonylglucoside. ^15^ Pelargonidin-3-glucoside. ^16^ Pelargonidin-3-malonylglucoside. ^17^ Pelargonidin-3-dimalonylglucoside. ^18^ Peonidin-3-glucoside. ^19^ Peonidin-3-malonylglucoside. ^20^ Peonidin-3-dimalonylglucoside. ^21^ Epicatechin-cyanidin-3,5-diglucoside. ^22^ Cyadinin-3-succinyl glucoside. ^23^ Cyadinin-3-ethyl malonyl glucoside. ^24^ Pelargonidin-3-diglucoside. ^25^ Cyanidin-3-disuccinyl glucoside. ^26^ Cyanidin-3-malonyl-succinyl glucoside. ^27^ Peonidin-3-succinyl glucoside.

**Table 3:** Other targeted metabolomic platforms used for the characterization of phenolic bioactive compounds in worldwide corn biodiversity.

Geographical Area	Type of Sample	Germplasm Origin (Place/Bank)	Analytical Configuration	Phenolic Metabolite in Mature Corn Kernels		Relevant Germplasm	Reference
				Free Fraction	Bound Fraction		
America	Landrace: Blue Bolita	Oaxaxa (Mexico)	HPLC-UV ^1^	Phenolic acids: syringic acid, chlorogenic acid, caffeic acid, vanillic acid, 4-hydroxybenzoic acid.	Phenolic acids: Ferulic acid, syringic acid, *p*-coumaric acid, chlorogenic acid.		[89]
	Twenty-two teosinte varieties: (*Zea perennis*, *Zea diploperennis*, *Zea nicagaraguensis*, *Zea luxurians*, *Zea mays spp. huehuetenangensis*, *Zea mays ssp. mexicana*, *Zea mays ssp. parviglumis*, Six modern corn varieties (*Zea mays ssp. mays*)	Banks: CIMMYT (International Maize and Wheat Improvement Center, Mexico). CINVESTAN (Centro de Investigación y de Estudios avanzados del Instituto Politécnico Nacional, Mexico) Corn seeds from Mexico, Nicaragua and Guatemala.	HPLC-DAD ^2^	Phenolic acids: *p*-coumaric acid, trans-ferulic acid.	Phenolic acids: *p*-coumaric acid, ferulic acid, diferulic acids (8-5’diferulic acid, 5-5’ diferulic acid).	Teosinte varieties (higher phenolic contents than commercial corn)	[90]
	Landraces: Elotero (blue), Chapalote (white)	Concordia (Sinaloa, Mexico)	HPLC-DAD ^2^	Phenolic acids: *p*-hydroxybenzoic acid, *p*-coumaric acid, sinapic acid, ferulic acid.	Phenolic acids: ferulic acid, *p*-coumaric acid, sinapic acid, vanillic acid, syringic acid, *p*-hydroxybenzoic acid.	White corn (high phenolic acid contents)	[91]
	Twenty-five blue hybrids derived from Criollo negro and Criollo Colorado landraces	Bank: INIFAP (National Forestry Agricultural and Husbandry Research Institute, Guanajuato, Mexico)	HPLC-DAD ^2^		Phenolic acid: ferulic acid	Hybrids with similar phenolic contents than landraces	[92]
	Eighteen corn phenotypes (commercial varieties and accessions)	Banks: CIMMYT (Mexico). Campo Cotaxtla (Mexico) Colegio de Postgraduados (Mexico)	HPLC-UV ^1^	Phenolic acids: ferulic acid Flavonoid: Anthocyanins	Phenolic acids: ferulic acid	AREQ516540TL and Veracruz 42 accessions high anthocyanin levels	[93]
	Twenty-two accessions from five landraces (Arequipeño, Kculli, Cabanita, Coruca, Granada.	Bank: Maize Research Program (Agraria University of La Molina, Peru)	UPLC-DAD ^3^	Phenolic acids: *p*-coumaric acid, *p*-coumaric acid derivatives, ferulic acid, ferulic acid derivatives, caffeic acid derivatives. Flavonols: quercetin derivatives. Anthocyanins.	*p*-coumaric acid, ferulic acid, ferulic acid derivatives	Kculli landrace (high phenolic contents)	[94]
America	Thirty-three accession from 14 local landraces	Bank: INIA (National Institute of Agronomic Research, La Platina, Chile)	HPLC-UV ^1^	Phenolic acids: vanillic acid, protocatecuic acid, *p*-coumaric acid, ferulic acid.	Phenolic acids: Ferulic acid, *p*-coumaric acid.	Pisankalla red landrace (high phenolic content)	[95]
	Landraces: Elote Occidental (red), Criollo Pozolero purpura (black), Cónico negro (black), Criollo amarillo. Vitamaize cultivars (blue/purple): E3E4, VMATF, VM366	Banks: Landraces (Michoacan, Mexico) Cultivars (CINVESTAV, Mexico)	Screening: DIESI-MSQD ^4^ Confirmation with: LC-MS/MS (QQQ) ^5^	Anthocyanins (18): Cy3G ^7^, Cy3MG ^8^, Cy3DMG ^9^, Cy3SG ^10^, Cy3DSG ^11^, Cy3MSG ^12^ Pg3G ^13^, Pg3MG ^14^, Pg3DMG ^15^, Pg3SG ^16^, Pg3DSG ^17^, Pg3MSG ^18^ Pn3G ^19^, Pn3MG ^20^, Pn3DMG ^21^, Pn3SG ^22^, Pn3DSG ^23^, Pn3MSG ^24^		Red (high in pelargonidin-based anthocyanins). Black (high in cyanidin derivatives)	[79]
	479 hybrids and 81 lines (White, yellow, red, blue, purple phenotypes)	Bank: Quantitative Genetics and Maize Breeding Program (Texas A&M University, United States)	FT-NIRS ^6^	Free soluble phenolic compounds		Well correlated with visual color selection for high anthocyanin corn	[80]
Europe	Landraces: Millo Corvo (blue), Scagliolo (yellow), Ottofile (yellow)	Blue corn (Galicia, Spain) Yellow corn (Careno and Zinasco, Italy)	HPLC-DAD ^2^	Anthocyanins: Cy3G, Pg3G, Pn3G		Millo Corvo (high anthocyanins)	[96]

^1^ High-performance liquid chromatography ultraviolet detector. ^2^ High-performance liquid chromatography diode array detector. ^3^ High-performance liquid chromatography diode array detector. ^4^ Direct injection electrospray ionization mass spectrometry. ^5^ Liquid chromatography triple quadrupole tandem mass spectrometry. ^6^ Fourier-transformed near-infrared reflectance spectroscopy. ^7^ Cyanidin-3-glucoside. ^8^ Cyanidin-3-malonyl glucoside. ^9^ Cyadinin-3-dimalonyl glucoside. ^10^ Cyanidin-3-succinyl glucoside. ^11^ Cyanidin-3-disuccinyl glucoside. ^12^ Cyanidin-3-malonyl succinyl glucoside. ^13^ Pelargonidin-3-glucoside. ^14^ Pelargonidin-3-malonyl glucoside. ^15^ Pelargonidin-3-dimalonyl glucoside. ^16^ Pelargonidin-3-succinyl glucoside. ^17^ Pelargonidin-3-disuccinyl glucoside. ^18^ Pelargonidin-3-malonyl succinyl glucoside. ^19^ Peonidin-3-glucoside. ^20^ Peonidin-3-malonyl glucoside. ^21^ Peonidin-3-dimalonyl glucoside. ^22^ Peonidin-3-succinyl glucoside. ^23^ Peonidin-3-disuccinyl glucoside. ^24^ Peonidin-3-malonyl succinyl glucoside.

**Table 4:** Targeted metabolomics for the characterization of carotenoid compounds in worldwide corn biodiversity.

Geographical Area	Type of Sample	Germplasm Origin (Place/Bank)	Analytical Configuration	Carotenoid Metabolite in Mature Corn Kernels		Relevant Germplasm	Reference
				Provitamin A	Non-provitamin A		
America	Landraces: Roxo, Palha Roxa, Mato Grosso, Palha Roxa, Rajado, Rajado 8 Carreiras, Roxo do Emilio, MPA, Língua de Papagaio	Santa Catarina (Brazil)	HPLC-UV-VIS ^1^	β-cryptoxanthin, α-carotene, cis-β-carotene, trans-β-carotene	Lutein, zeaxanthin	Palha Roxa, MPA, Roxo (high contents)	[102]
	Twenty-six landraces (yellow, white, orange, variegated, purple)	SINTRA-Small Farmer Association (Santa Catarina, Brazil)	HPLC-UV-VIS ^1^	β-cryptoxanthin, α-carotene, β-carotene.	Lutein, zeaxanthin	Roxo 41 and MPA1 (high contents)	[103]
	Twenty-two landraces (white, yellow, orange)	Brazil (Bank not informed)	HPLC-DAD ^2^	β-cryptoxanthin, α-cryptoxanthin, α-carotene, β-carotene.	Lutein, zeaxanthin	MC3, MC14 (high contents)	[104]
	Eight landraces: Tuxpeño (yellow), Tablocillo (red), Chapalote (red)	Sinaloa (Mexico)	HPLC-DAD ^2^	β-cryptoxanthin, β-carotene	Lutein, zeaxanthin	Tuxpeño (high contents)	[105]
Africa	Landraces: 26 white and 35 orange (*Mthikinya*)	Central Malawi	HPLC-DAD ^2^	β-cryptoxanthin, β-carotene	Lutein, zeaxanthin	Orange group with high contents	[106]
	421 tropical adapted yellow endosperm inbred lines	Ibadan (Nigeria)	HPLC-DAD ^2^	β-cryptoxanthin, α-carotene, β-carotene (cis+trans isomers)	Lutein, zeaxanthin	Variable	[107]
Asia	Sweet corn varieties: Jingtian 3, Jingtian 5. Waxy corn varieties: Suyunuo 11, Jignuo 8, Jingtianzihuanuo 2	China (Bank not informed)	HPLC-DAD-APCI-MS/MS ^3^	All-trans-α-carotene, 9-cis-α-carotene, 9′-cis-α-carotene, all-trans-β-carotene, 9-cis-β-carotene, 13-cis-β-carotene, all-trans-β-cryptoxanthin, 9-cis-β-cryptoxanthin, 9′-cis- β-cryptoxanthin, 13 or 13’-cis- β-cryptoxanthin, 15-cis- β-cryptoxanthin, all-trans-α-cryptoxanthin, 9-cis-α-cryptoxanthin	All-trans-lutein, 9 or 9′-cis-lutein, 13-cis-lutein-5,6-epoxide, all-trans-zeaxanthin, violaxanthin, neochrome, neoxanthin, 13-cis-neoxanthin	Sweet varieties (high contents)	[98]
	Sweet corn varieties: Jingtian 5, Suyu 29	Luhe Experimental Station of Jiangsu Academy of Agricultural Sciences (China)	HPLC-DAD-APCI-MS/MS ^3^	All-trans-α-carotene, all-trans-β-carotene, all-trans-β-cryptoxanthin, all-trans-α-cryptoxanthin	Neoxanthin, violaxanthin, all-trans-lutein, all-trans-zeaxanthin.	Suyu 29 with orange kernel (high contents)	[108]
Europe	Four landrances: Formentone ottofile rosso (red), Formentone ottofile giallo (yellow), Nostrato del Palazzaccio (yellow), Nano di Verni (yellow), yellow comercial variety.	Regional Germplasm Bank Network (Italy). Comunitá Montana della Mediavalle del Serchio (Italy)	HPLC-DAD ^2^	β-cryptoxanthin, β-carotene.	Lutein, zeaxanthin	Nano di Verni (high contents)	[109]
	93 Landraces	European Union Maize Landraces Core Collection (EUMLCC)	HPLC-DAD ^2^		Lutein, zeaxanthin	Overall landraces from Italy and France (high contents)	[110]
Mixed	Ten landraces and inbred lines	CYMMIT (International Maize and Wheat Improvement Center, Mexico). BSSS (Iowa Stiff Stalk Syntetic, United States). MRI (Maize Research Institute, Serbia). Samples from Mexico, United States, France, Serbia, Netherland.	HPLC-DAD ^2^	β-carotene	Lutein	Orange and red colored corn kernels (high contents)	[111]

^1^ High-performance liquid chromatography ultraviolet-visible detector. ^2^ High-performance liquid chromatography diode array detector. ^3^ High-performance liquid chromatography diode array detector atmospheric pressure chemical ionization tandem mass spectrometry.

**Table 5:** Investigations about the corn genetic diversity using different types of markers.

Germplasm	Origin	Evaluated Trait	Type of Marker	Method for Diversity Classification	Reference
Eight Peruvian highland corn landraces: Confite Morocho (5) ^1^, Chullpi (6), Uchuquilla (5), Cusco Gigante (9), Huayleño (9), Paro (4), San Gerónimo-Huancavelicano (6), Shajatu (6)	Peru	Plant traits: height, ear height, leaf number, leaf number above ear, leaf length, leaf width	Morphological	Ward-Modified Location Model (MLM)	[123]
Four Peruvian highland corn landraces: Confite Morocho (5) ^1^, Confite Punteagudo (9), Cusco Gigante (5), Uchuquilla (5)	Peru	Internal ear traits: cob and pith diameters, glume length and texture, cupule length and width	Morphological	Ward-MLM	[124]
134 corn populations from 34 highland localities and 10 witnesses	Mexico	32 vegetative, reproductive, and yield traits	Morphological	Modified Location Model (MLM)	[131]
Thirty-four accessions (Choclero landrace)	Chile	2 phenological, 16 vegetative 12 reproductive and 9 qualitative traits	Morphological	Ward and Manhattan distance method	[125]
Seventy-nine corn accessions: flint (59) ^1^, pop (16) and dent (4) races	Turkey	16 traits: 6 ear and 10 tassel traits	Morphological	Unweighted pair group method of arithmetic average (UPMGA)	[126]
Six corn landraces: Altiplano (32) ^1^, Blanco (13), Amarillo Grande (25), Amarillo Chico (34), Pisingallo (16) and Orgullo Cuarentón (24)	Argentina	---	Molecular: 18 SSR (microsatellites or simple sequence length polymorphisms)	Bayesian method and Nei’s genetic distances	[128]
Corn inbred lines: group I: 7 tropical and subtropical lines; group II: 7 temperate lines	Group I: Brazil Group II: United States	---	Molecular: 22 SSR	Markov Chain Monte Carlo algorithm for the Bayesian clustering method and the Self-Organizing Tree Algorithm (SOTA)	[127]
Ninety white dent race accessions	Uruguay	17 traits: vegetative, reproductive and yield	Morphological Molecular: 26 SSR	Molecular results: Ward, canonical and Bayesian methods	[129]
349 inbred lines: 283 ex-PVP (Plant Variety Protected) inbreds and 66 public inbred lines.	United States	---	Sequence-based markers: Genotyping-by-sequencing (GBS), 77,314 SNP (single-nucleotide polymorphism) markers	Ward and Nei’s distance methods	[130]

^1.^ Number of accessions.
